# Low-Density Lipoprotein Cholesterol, Type 2 Diabetes and Progression of Aortic Stenosis: The RED-CARPET Heart Valve Subgroup Cohort Study

**DOI:** 10.31083/j.rcm2508276

**Published:** 2024-08-07

**Authors:** Jingjing He, Zhenyu Xiong, Odong Christopher, Zhuoshan Huang, Chaoguang Xu, Menghui Liu, Miaohong Li, Zhen Guo, Xinxue Liao, Xiaodong Zhuang

**Affiliations:** ^1^Cardiology Department, First Affiliated Hospital of Sun Yat-sen University, 510080 Guangzhou, Guangdong, China; ^2^NHC Key Laboratory of Assisted Circulation (Sun Yat-sen University), 510275 Guangzhou, Guangdong, China; ^3^Cardiology Department, The Third Affiliated Hospital of Sun Yat-sen University, 510630 Guangzhou, Guangdong, China

**Keywords:** aortic stenosis, progression rate, type 2 diabetes mellitus, LDL-C, risk factors

## Abstract

**Background:**

Low-density lipoprotein cholesterol (LDL-C) and type 2 diabetes (T2DM) are both independent risk factors for aortic stenosis (AS). In AS patients, whether LDL-C or T2DM is associated with fast AS progression (FASP) and their interaction is unknown. This study aims to test the hypothesis that there is a heightened risk of FASP when elevated LDL-C coexists with T2DM.

**Methods:**

The Real-world Data of Cardiometabolic Protections (RED-CARPET) study enrolled participants with mild (peak aortic velocity = 2–3 m/s), moderate (3–4 m/s) and severe (≥4 m/s) AS between January 2015 and December 2020 at a single center. Participants were further stratified by baseline LDL-C joint T2DM, follow-up echocardiography was performed after 6 months, and the primary outcome was FASP, defined as the annual change in aortic peak velocity (≥0.3 m/s/year).

**Results:**

Among the 170 participants included, 45.3% had mild AS, 41.2% had moderate AS, and 13.5% had severe AS. The mean age was 66.84 ± 12.64 years, and 64.1% were women. During the follow-up period of 2.60 ± 1.43 years, 35 (20.6%) cases of FASP were identified. Using non-T2DM with LDL-C <2.15 mmol/L as reference, FASP risk was 1.30 [odds ratio (OR), 95% CI (0.99–7.78, *p* = 0.167)] for non-T2DM with LDL-C 2.15–3.14 mmol/L, 1.60 [OR, 95% CI (1.17–3.29, *p* = 0.040)] for non-T2DM with LDL-C ≥3.14 mmol/L, 2.21 [OR, 95% CI (0.49–4.32, *p* = 0.527)] for T2DM with LDL-C <2.15 mmol/L, 2.67 [OR, 95% CI (1.65–7.10, *p* = 0.004)] for T2DM with LDL-C 2.15–3.14 mmol/L, and 3.20 [OR, 95% CI (1.07–5.34, *p* = 0.022)] for T2DM with LDL-C ≥3.14 mmol/L.

**Conclusions:**

LDL-C joint T2DM was associated with FASP. This investigation suggests that fast progression of AS may develop if LDL-C is poorly managed in T2DM. Additional research is needed to validate this finding and explore the possible biological mechanism to improve the cardiometabolic management of T2DM and seek possible prevention for AS progression for this population.

**Clinical Trial Registration:**

ChiCTR2000039901 (https://www.chictr.org.cn).

## 1. Introduction

Type 2 diabetes mellitus (T2DM) is a prevalent chronic disease that poses a significant threat to populational health. Approximately 30%–40% of the T2DM population also has aortic stenosis (AS) [1], which is the second most common valvular heart disease in developed countries, especially among people over the age of 65 years [2], and carries a poor prognosis at a severe stage if not treated by valve replacement. AS and T2DM are both silent chronic progressive diseases that result in significant cardiovascular morbidity and mortality in developed countries [1, 3]. Furthermore, AS and T2DM are anticipated to increase gradually due to an aging population, increasing lipid abnormalities and the obesity pandemic [2, 4]. Understanding AS progression and associated factors in this vulnerable group of patients afflicted with both diseases represents an opportunity to improve cardiovascular outcomes and optimize care.

From previous investigations, the development of AS is considered a degenerative process due to the accumulation of wear and tear, leading to passive calcium deposition [5, 6, 7, 8]. AS is characterized by progressive pathological calcification defects in the cusps of the aortic valve leaflets [9, 10], identical to the progression mechanism in coronary heart disease (CHD) [11], which causes the leaflets to become thick, stiff, and calcified. However, recent compelling evidence has argued otherwise, suggesting that AS is an active and multifactorial disease involving numerous atherosclerotic pathophysiological pathways [12, 13, 14]. In this regard, well-known atherosclerotic risk factors, including age, sex, smoking, hypertension, hypercholesterolemia, obesity, metabolic syndrome, diabetes mellitus, and elevated plasma levels of lipoprotein(a) (Lp[a]) and low-density lipoprotein cholesterol (LDL-C), have been correlated with the development and/or progression of AS [15, 16, 17]. Among these, diabetes and dyslipidemia were each independently associated with the incidence AS, with its significance ranking just after hypertension [18].

However, while these studies strengthened our understanding of links between these diseases and provided insight into AS prevention, they gave little information in terms of clinical management in those with T2DM who already have AS, which constitutes a considerable representation in clinical practice [19, 20, 21]. As a major cardiovascular risk factor, lipid management is one of the pillars in the contemporary multifaceted approach to reducing T2DM complications [22]. Previous research found that LDL-C in T2DM is commonly elevated [23], and its atherogenic lipid phenotype is characterized by small, dense LDL-C particles that contribute to the more rapid development and progression of coronary atherosclerosis [24, 25]. What these elevated LDL-C levels indicate in terms of the risk of AS progression in T2DM patients has not been researched. While current guidelines set specific LDL-C goals for T2DM patients with varying cardiovascular risk strata [22, 26, 27], the question remains how LDL-C should be controlled in those also suffering from AS, due to a gap in evidence. This study aims to test the hypothesis that in T2DM patients with AS, T2DM itself combined with higher serum LDL-C levels is associated with fast aortic stenosis progression (FASP).

## 2. Materials and Methods

### 2.1 Study Design and Population

The Real-world Data of Cardiometabolic Protections (RED-CARPET) heart valve subgroup study is an ongoing cohort study of participants aged ≥18 yers with CHD, hypertension (HTN), T2DM, dyslipidemia or valvular heart disease recruited during hospitalization at the cardiovascular unit of the First Affiliated Hospital of Sun Yat-sen University in China. For this investigation, we enrolled participants with well-established AS from 1 January 2015 to 30 December 2020. Follow-up was performed by telephone or questionnaires, and echocardiography was conducted after 6 months (any timepoint after 6 months, with varying frequency). Among the participants recruited, peak aortic velocity (Vmax) was categorized as mild (2.0–3.0 m/s), moderate (3.0–4.0 m/s), and severe (≥4.0 m/s) AS. If a patient completed multiple echocardiography measurements, the latest or preoperational Vmax was selected and included in the analysis.

We excluded participants with rheumatic heart disease, missing demographic information including body mass index (BMI), alcohol consumption, smoking, LDL-C, glucose (GLU), high-density lipoprotein cholesterol (HDL-C), total cholesterol (TC), creatinine (Cr), uric acid (UA), comorbidities such as hypertension, T2DM, CHD, and incomplete cardiac echocardiogram data.

#### 2.1.1 Laboratory Lipid Measurements

For each participant, fasting blood samples were obtained after 12 hr of fasting upon admission. Blood samples were collected into an ethylene diamine tetraacetic acid (EDTA)-containing tube. After centrifugation at 3000 rpm for 10 min at 4 °C, plasma was collected and stored at –80 °C. An automatic biochemistry analyzer measured the plasma concentrations of TC, triglycerides (TG), LDL-C (cutoff value ≥2.6 mmol/L in men and ≥3.5 mmol/L in females) and HDL-C (cutoff value ≥1 mmol/L in men and ≥1.3 mmol/L in females) in an enzymatic assay (Hitachi 150, Tokyo, Japan) [28].

#### 2.1.2 Echocardiography

Available doppler-echocardiograms for each participant were gathered from the hospital information system. Trans-thoracic echocardiography was performed using commercially available ultrasound systems in the left lateral decubitus position. Board certified sonographers performed all the examinations using uniform equipment (Philips iE33 Ultrasound systems, Philips Healthcare, Amsterdam, North Holland, Netherlands).

#### 2.1.3 Definition of Aortic Stenosis and Type 2 Diabetes Mellitus

The severity of AS was defined according to current guidelines as follows: mild (Vmax ≥2.0–3.0 m/s, aortic valve area [AVA] ≥1.5 ${\mathrm{cm}}^{2}$, mean pressure gradient [MPG] <20 mmHg), moderate (Vmax ≥3.0–4.0 m/s, AVA 1.0–1.5 ${\mathrm{cm}}^{2}$, MPG 20–40 mmHg), and severe (Vmax ≥4.0 m/s, AVA <1.0 ${\mathrm{cm}}^{2}$, MPG ≥40 mmHg) [29, 30].

T2DM was diagnosed as fasting blood glucose ≥7.0 mmol/L, nonfasting blood glucose ≥11.1 mmol/L, glycated hemoglobin ≥6.5%, a random blood sugar test of ≥11.1 mmol/L with associated symptoms and use of antidiabetic medicines, or self- or physician-reported diagnosis [2].

#### 2.1.4 Progression of Aortic Stenosis

The progression rate of AS was calculated from the change in Vmax. Vmax1 was defined as Vmax at baseline, and Vmax2 was defined as Vmax at follow-up. The AS progression rate was calculated for each person using the following formula [30, 31]:

Hemodynamic progression rate of ⁢AS=Vmax2-Vmax1 Follow -up⁢Time⁡(T)

The fast progression rate of AS was defined by the 2018 document endorsed by the European Association of Cardiovascular Imaging and the American Society of Echocardiography as annual progression rates ≥0.3 m/s/year, and slow or no progression was defined as progression rates <0.3 m/s/year [30, 31, 32].

### 2.2 Statistical Analysis

Demographic characteristics were described by FASP and further stratified by LDL-C joint T2DM. The categorical clinical characteristics are presented as counts (percentage), continuous variables are presented as the mean ± standard deviation, and categorical variables were compared using the chi-square test. Continuous variables were compared using the unpaired Student’s *t* test or the Mann‒Whitney U test depending on variable distribution between slow/no AS progression and FASP and one-way analysis of variance (ANOVA) or the Kruskal‒Wallis test as appropriate among T2DM joint LDL-C subgroups. We also separately analyzed the risk of FASP by LDL-C levels and the presence/absence of T2DM.

Covariates include characteristics that might confound outcomes, including demographics (age, sex, BMI, UA, Cr, smoking, alcohol intake, systolic and diastolic blood pressure) and comorbidities. Multivariable logistic regression models were conducted among T2DM joint LDL-C subgroups distributed as group 1: non-T2DM+LDL-C <2.15 mmol/L, group 2: non-T2DM+LDL-C 2.15–3.14 mmol/L, group 3: non-T2DM+LDL-C ≥3.14 mmol/L, group 4: T2DM+LDL-C <2.15 mmol/L, group 5: T2DM+LDL-C 2.15–3.14 mmol/L, group 6: T2DM+LDL-C ≥3.14 mmol/L, for each participant to estimate the odds ratio (OR) and 95% confidence intervals (95% CIs) for FASP risk. Using non-T2DM patients with LDL-C <2.15 mmol/L as the reference, we constructed three models to control for confounding variables and evaluated the association between LDL-C joint T2DM and FASP; model-1 was unadjusted; model-2 was partially adjusted for age and sex; and model-3 was fully adjusted for model-2 plus BMI, TC, Cr, HTN and CHD. All statistical analyses were performed using SPSS version 25.0 (SPSS Inc., IBM, Chicago, IL, USA). *p* values are two-sided, and *p* values < 0.05 and 95% CIs were regarded as statistically significant.

## 3. Results

### 3.1 Baseline Characteristics

A total of 170 participants were followed up from 1 January 2015 to 30 December 2020; 64.1% were women, and the mean age was 66.84 ± 12.64 years; 45.3% had mild AS, 41.2% had moderate AS, and 13.5% had severe AS. During a follow-up period of 2.60 ± 1.43 years, 35 (20.6%) cases of FASP were identified. Participants with FASP were slightly older, were more frequently male, had a mean BMI of 22.94 ± 3.48 kg/$m^{2}$, had a serum Cr level of 97.78 ± 40.32 µmol/L, had a higher prevalence of CHD and T2DM, and had numerically higher LDL-C. On the other hand, T2DM patients with LDL-C ≥3.14 mmol/L were slightly older, male, and had a BMI of 25.41 ± 4.48 kg/$m^{2}$ (Tables 1,2).

**Table 1.** **Study sample characteristic stratified by annual progression rate of aortic stenosis**.

Variables		Total	Slow or no AS progression	Fast AS progression	*p* value
		(n = 170)	n = 135 (79.4%)	n = 35 (20.6%)	
Age, yr		66.84 ± 12.64	65.64 ± 11.52	71.46 ± 15.61	0.005
Male, n (%)		61 (35.9)	43 (31.8)	18 (51.4)	0.031
BMI, kg/$m^{2}$		26.99 ± 6.43	28.04 ± 6.60	22.94 ± 3.48	<0.001
Alcohol intake, n (%)					0.466
	Never	137 (80.6)	111 (82.2)	26 (74.3)	
	Ever	32 (18.8)	24 (17.8)	8 (22.8)	
Smoking, n (%)					0.098
	Never smoked	138 (81.2)	113 (83.7)	25 (71.4)	
	Ever smoked	32 (18.8)	22 (16.3)	10 (28.6)	
SBP, mmHg		132.80 ± 21.10	132.98 ± 21.02	132.11 ± 21.70	0.618
DBP, mmHg		75.22 ± 12.88	73.71 ± 13.05	75.61 ± 12.86	0.579
PP, mmHg		57.58 ± 16.91	57.36 ± 15.62	58.40 ± 21.42	0.870
GLU, mmol/L		6.01 ± 1.93	6.13 ± 1.98	5.54 ± 1.64	0.063
TC, mmol/L		4.75 ± 1.31	4.82 ± 1.12	4.74 ± 1.36	0.737
TG, mmol/L		1.29 ± 0.87	1.26 ± 0.84	1.43 ± 0.95	0.278
HDL-C, mmol/L		1.25 ± 0.39	1.27 ± 0.40	1.18 ± 0.31	0.195
LDL-C, mmol/L		3.00 ± 0.99	2.97 ± 1.05	3.10 ± 0.76	0.231
Cr, µmol/L		103.13 ± 108.66	104.51 ± 120.28	97.78 ± 40.32	0.027
UA, µmol/L		431.19 ± 141.77	434.36 ± 143.45	418.96 ± 136.38	0.482
HTN, n (%)		111 (65.3)	92 (68.1)	19 (54.3)	0.163
T2DM, n (%)		44 (25.9)	30 (22.2)	14 (40.0)	0.030
CHD, n (%)		43 (25.3)	27 (20.0)	16 (45.7)	0.002
Aortic stenosis, n (%)					0.780
	Mild	77 (45.3)	62 (45.9)	15 (42.9)	
	Moderate	70 (41.2)	56 (41.5)	14 (40.0)	
	Severe	23 (13.5)	17 (12.6)	6 (17.1)	
ΔVmax, m/s		0.38 ± 0.87	0.14 ± 0.43	1.34 ± 1.36	<0.001
Vmax, m/s		0.38 ± 0.87	3.20 ± 0.77	3.34 ± 0.91	0.356
PG, mmHg		33.47 ± 21.00	33.30 ± 20.97	34.11 ± 21.44	0.348
EF, %		63.55 ± 10.56	64.03 ± 10.14	61.71 ± 12.04	0.249
Follow-up, months, median (interquartile range)		30.7 (17.9, 41.3)	34.9 (19.3, 43.0)	24.0 (10.1, 31.9)	<0.01

Data are median (interquartile range), mean ± SD, or percentage (unless otherwise indicated). According to AS annual progression rate, characteristics are from the study population (n = 170). BMI, body mass index; SBP, systolic blood pressure; DBP, diastolic blood pressure; PP, pulse pressure; GLU, glucose; TC, total cholesterol; TG, triglycerides; HDL-C, high-density lipoprotein cholesterol; LDL-C, low-density lipoprotein cholesterol; AS, aortic stenosis; Cr, serum creatinine; UA, uric acid; HTN, hypertension; T2DM, type 2 diabetes mellitus; CHD, coronary heart diseases; ΔVmax, m/s/year, annual change in peak aortic velocity; PG, pressure gradient; EF, ejection fraction.

**Table 2.** **Participants baseline characteristic stratified by T2DM joint LDL-C**.

Variables		Group 1 (n = 30)	Group 2 (n = 41)	Group 3 (n = 55)	Group 4 (n = 13)	Group 5 (n = 15)	Group 6 (n = 16)	*p*-value
Age, yr		71.17 ± 13.81	66.49 ± 12.27	63.13 ± 12.19	69.69 ± 15.00	69.40 ± 12.01	67.63 ± 8.82	0.084
Male		14 (46.7)	16 (39.0)	16 (29.1)	3 (23.1)	6 (40.0)	6 (37.5)	0.571
BMI, kg/$m^{2}$		26.62 ± 6.85	27.02 ± 7.70	28.19 ± 6.42	25.84 ± 4.60	25.94 ± 4.71	25.41 ± 4.48	0.583
Alcohol intake, n (%)								0.475
	Never	25 (83.3)	31 (75.6)	42 (76.4)	12 (92.3)	14 (93.3)	13 (81.2)	
	Ever	5 (16.7)	10 (24.4)	13 (23.6)	1 (7.7)	1 (6.7)	2 (12.5)	
Smoking, n (%)								0.988
	Never smoked	23 (76.7)	34 (82.9)	45 (81.8)	11 (84.6)	12 (80.0)	13 (81.2)	
	Ever smoked	7 (23.3)	7 (17.1)	10 (18.2)	2 (15.4)	3 (20.0)	3 (18.7)	
SBP, mmHg		125.53 ± 18.53	131.83 ± 18.71	137.95 ± 21.77	137.77 ± 22.46	127.67 ± 22.46	132.00 ± 24.19	0.127
DBP, mmHg		70.10 ± 14.36	75.32 ± 10.04	76.40 ± 10.88	79.38 ± 21.88	76.00 ± 15.52	76.44 ± 9.64	0.239
PP, mmHg		55.43 ± 13.79	56.51 ± 14.63	61.55 ± 20.24	58.38 ± 15.35	51.67 ± 14.84	55.56 ± 17.40	0.332
GLU, mmol/L		5.89 ± 1.80	5.54 ± 1.03	5.50 ± 1.16	6.66 ± 2.42	6.77 ± 1.81	7.94 ± 3.71	<0.001
TC, mmol/L		3.36 ± 0.95	4.36 ± 0.64	5.93 ± 0.89	3.46 ± 0.60	4.22 ± 0.39	5.88 ± 0.87	<0.001
TG, mmol/L		1.24 ± 1.43	1.07 ± 0.49	1.45 ± 0.86	1.15 ± 0.40	1.19 ± 0.43	1.67 ± 0.68	0.138
HDL-C, mmol/L		1.25 ± 0.53	1.29 ± 0.41	1.31 ± 0.33	1.15 ± 0.38	1.13 ± 0.22	1.16 ± 0.30	0.402
LDL-C, mmol/L		1.83 ± 0.27	2.63 ± 0.30	4.01 ± 0.62	1.97 ± 0.13	2.62 ± 0.26	3.87 ± 0.66	<0.001
Cr, µmol/L		91.68 ± 44.76	105.04 ± 138.82	87.91 ± 59.52	76.05 ± 22.06	170.65 ± 217.74	130.70 ± 115.04	0.105
UA, µmol/L		399.04 ± 155.02	400.23 ± 144.07	443.35 ± 124.27	427.16 ± 143.61	498.84 ± 171.52	468.86 ± 117.21	0.131
HTN, n (%)		20 (66.7)	26 (63.4)	34 (61.8)	9 (69.2)	12 (80.0)	10 (6.2)	0.855
DM, n (%)		0 (0.0)	0 (0.0)	0 (0.0)	13 (100.0)	15 (100.0)	16 (100.0)	<0.001
CHD, n (%)		9 (30.0)	5 (12.2)	12 (21.8)	5 (38.5)	6 (40.0)	6 (37.5)	0.126
AS, n (%)								0.006
	Mild	11 (36.7)	15 (36.6)	24 (43.6)	7 (53.8)	6 (40.0)	14 (87.5)	
	Moderate	11 (36.7)	21 (51.2)	28 (50.9)	4 (30.8)	5 (33.3)	1 (6.2)	
	Severe	8 (26.7)	5 (12.2)	3 (5.4)	2 (15.4)	4 (26.7)	1 (6.2)	
Vmax, m/s		3.50 ± 0.89	3.34 ± 0.77	3.22 ± 0.68	3.00 ± 0.75	3.35 ± 0.84	2.56 ± 0.75	0.004
PG, mmHg		43.30 ± 27.23	31.24 ± 18.08	31.31 ± 19.54	34.92 ± 20.83	40.53 ± 21.56	20.38 ± 7.19	0.007
EF, %		61.50 ± 11.22	64.02 ± 10.19	65.11 ± 10.90	63.39 ± 7.60	62.60 ± 11.08	61.88 ± 11.23	0.715
Follow-up, months, median (interquartile range)		36.0 (19.2–49.2)	28.8 (13.2–38.4)	34.8 (20.4–42.0)	18.0 (13.2–40.8)	31.2 (19.2–40.8)	32.4 (14.4–44.4)	0.600

Data are median (interquartile range), mean ± standard deviation, or percentage (unless otherwise indicated). According to AS annual progression rate, variables are from the study population of n = 170; group 1 = non-T2DM+LDL-C <2.15 mmol/L, group 2 = non-T2DM+LDL-C 2.15–3.14 mmol/L, group 3 = non-T2DM+LDL-C ≥3.14 mmol/L, group 4 = T2DM+LDL-C <2.15 mmol/L, group 5 = T2DM+LDL-C 2.15–3.14 mmol/L, group 6 = T2DM+LDL-C ≥3.14 mmol/L. BMI, body mass index; SBP, Systolic blood pressure; DBP, diastolic blood pressure; PP, pulse pressure; GLU, glucose; TC, total cholesterol; TG, triglycerides; HDL-C, high-density lipoprotein cholesterol; LDL-C, low-density lipoprotein cholesterol; AS, aortic stenosis; Cr, serum creatinine; UA, uric acid; HTN, hypertension; T2DM, type 2 diabetes mellitus; DM, diabetes mellitus; CHD, coronary heart diseases; Vmax, peak aortic velocity; PG, mean pressure gradient; EF, ejection fraction.

### 3.2 LDL-C, T2DM and Fast Aortic Stenosis Progression Rate

Overall, 17.6% were non-T2DM with LDL-C <2.15 mmol/L (group 1), 24.1% were non-T2DM with LDL-C 2.15-3.14 mmol/L (group 2), 32.4% were non-T2DM with LDL-C ≥3.14 mmol/L (group 3), 7.6% were T2DM with LDL-C <2.15 mmol/L (group 4), 8.8% were T2DM with LDL-C 2.15-3.14 mmol/L (group 5), and 9.4% were T2DM with LDL-C ≥3.14 mmol/L (group 6) (Table 2).

**Fig. 1.**
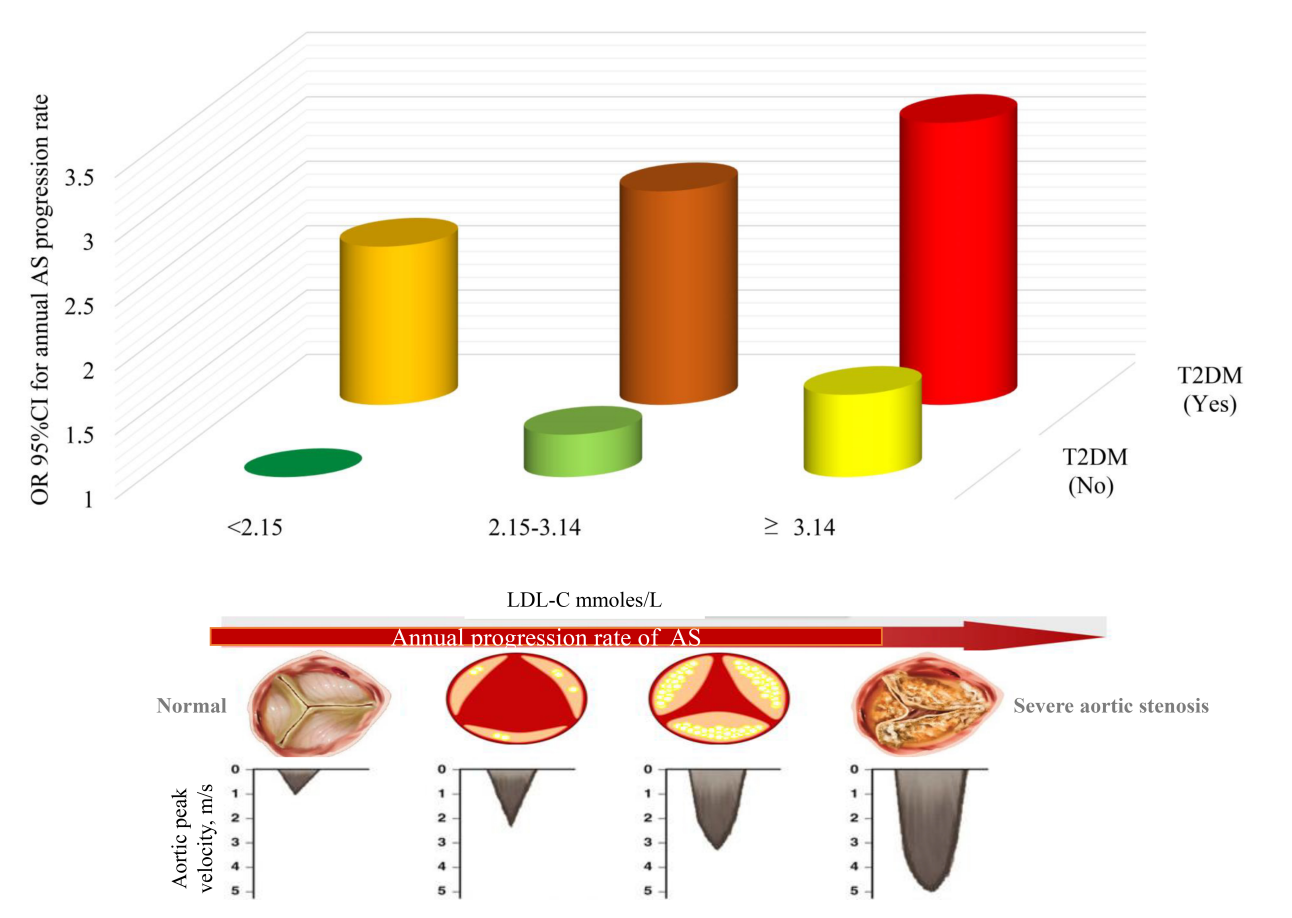
**Risk of fast aortic stenosis progression stratified by LDL-C joint T2DM, adjusted for age, sex, body mass index, total cholesterol, creatinine, hypertension, and coronary heart disease**. LDL-C, low-density lipoprotein cholesterol; T2DM, type 2 diabetes mellitus; OR, odds ratio; CI, confidence interval; AS, aortic stenosis.

Using group 1 as the reference group, FASP risk was 1.30 (OR, 95% CI 0.99–7.78, *p* = 0.167) for group 2, 1.60 (OR, 95% CI 1.17–3.29, *p* = 0.040) for group 3, 2.21 (OR, 95% CI 0.49–4.32, *p* = 0.527) for group 4, 2.67 (OR, 95% CI 1.65–7.10, *p* = 0.004) for group 5, and 3.20 (OR, 95% CI 1.07–5.34, *p* = 0.022) for group 6. The P for the interaction between LDL-C and T2DM categorical subgroups was 0.021 (Table 3, Fig. 1).

**Table 3.** **Adjusted OR for fast progression rate of aortic stenosis associated LDL-C joint T2DM**.

Exposure T2DM	LDL-C, mmol/L	Total (n)	Unadjusted		Partially adjusted model		Fully adjusted model	
		n = 170	OR (95% CI)	*p* value	OR (95% CI)	*p* value	OR (95% CI)	*p* value
		n (%)						
No	<2.15	30 (17.6)	Ref = 1					
	2.15–3.14	41 (24.1)	4.70 (0.91–8.36)	0.208	1.20 (0.96–6.55)	0.088	1.30 (0.99–7.78)	0.167
	≥3.14	55 (32.4)	2.79 (0.52–5.05)	0.089	1.56 (1.21–2.02)	0.017	1.60 (1.17–3.29)	0.040
Yes	<2.15	13 (7.6)	2.46 (0.30–3.68)	0.379	2.10 (1.57–2.79)	0.251	2.21 (0.49–4.32)	0.527
	2.15–3.14	15 (8.8)	3.64 (1.53–4.63)	0.013	2.21 (1.96–5.57)	0.006	2.67 (1.65–7.10)	0.004
	≥3.14	16 (9.4)	1.75 (1.04–8.74)	0.018	2.59 (1.17–6.40)	0.005	3.20 (1.07–5.34)	0.022
*p* for interaction								0.021

Unadjusted model; Model 1-partially adjusted for age and gender; Fully adjusted model adjusted for model-1 plus BMI, TC, Cr, HTN and CHD. OR, odds ratio; CI, confidence interval; LDL-C, low-density lipoprotein cholesterol; T2DM, type 2 diabetes mellitus; BMI, body mass index; TC, total cholesterol; Cr, creatine; HTN, hypertension; CHD, coronary heart disease.

Separately, the risk of FASP was 2.42 (OR, 95% CI 1.00–5.584, *p* = 0.050) among T2DM patients compared with non-diabetic patients. Among the LDL-C tertiles (<2.15, 2.15–3.14, and ≥3.14 mmol/L) subgroups, the risk of FASP was 5.42 (OR, 95% CI 1.24–8.80, *p* = 0.025) among the LDL-C <2.15 mmol/L subgroup and 11.90 (OR, 95% CI 1.55–15.65, *p* = 0.017) among the LDL-C ≥3.14 mmol/L subgroup, using participants with LDL-C <2.15 mmol/L as the reference (**Supplementary Table 1**).

## 4. Discussions

No consensus or recommendation exists for managing serum LDL-C levels in T2DM patients with well-established AS. Our study responds to this gap by reporting that among AS patients, non-T2DM with LDL-C ≥3.14 mmol/L was associated with a 1.6-fold higher risk of FASP, T2DM with LDL-C 2.15–3.14 mmol/L was associated with a 2.7-fold higher risk and T2DM with LDL-C ≥3.14 was associated with a 3.2-fold higher risk of FASP. This is the first prospective cohort study to assess the association of LDL-C and T2DM with the risk of FASP.

Previous investigations have shown that high serum LDL-C is commonly present among T2DM patients and is correlated with CHD morbidity and mortality through the acceleration of the atherosclerotic process [33]. Hence, current clinical practice guidelines recommend aggressive LDL-C level management in T2DM [26], especially for those with established CHD. However, whether LDL-C influences AS progression and prognosis in T2DM patients is unclear. There is a gap in guidelines in terms of appropriate LDL-C management in T2DM patients for those with well-established AS. Our investigation stratified tertiles of LDL-C joint T2DM and found that the risk of FASP was highest among T2DM patients with LDL-C levels ≥3.14 mmol/L.

Our finding is consistent with the findings of Robinson and Stone [25], who reported that AS is an active and multifactorial disease and shares numerous pathophysiological backgrounds with atherosclerosis that are commonly associated with T2DM patients with elevated LDL-C or well-established CHD and T2DM risk factors. We also found that elevated LDL-C levels were associated with FASP, consistent with the study by Pérez *et al*. [34], whose investigations suggested that a reduction in LDL-C and Lp(a) could mitigate the progression of AS and that elevated LDL-C increased the need for aortic valve surgery. Regardless, this is contradicted by the 2020 clinical guidelines for the management of patients with valvular heart disease, which firmly suggested a limited influence of LDL-C on AS progression and did not endorse statins as a treatment of choice to restrict or slow AS progression due to limited evidence [29]. Of note, this study reports a significant interaction (*p* = 0.021) between LDL-C and T2DM subgroups, suggesting a significant influence of serum LDL-C levels in stimulating fast AS progression in T2DM patients with well-established AS, which could be explained by the theory that T2DM patients with elevated LDL-C may have more active and pronounced atherosclerotic pathophysiological processes, including inflammation and cell calcification [24, 25].

It is well known that AS and T2DM are both chronic progressive diseases common in the elderly and may result in significant mortality if left untreated [35]. Following the growth of obesity and widespread aging, the incidence of AS and T2DM is expected to increase [2, 4]. As a considerable portion of T2DM patients also have AS, a vicious disease with poor prognosis at a severe stage, understanding the prevention of AS progression in this population is of tremendous medical and socioeconomic significance [36]. Previous studies have demonstrated that LDL-C and T2DM are well-established surrogates and independent CHD and AS risks [33] and have separately explored the association between AS, LDL-C and T2DM [37]. However, considerable contradictions still exist [36, 38]. We focused our investigation on FASP risk in participants stratified by LDL-C joint T2DM using the RED-CARPET cohort study. Interestingly, patients with FASP actually had marginally lower blood glucose (6.13 ± 1.98 vs 5.54 ± 1.64, *p* = 0.063), and while the 6 groups stratified by T2DM joint LDL-C produced significantly different glucose levels, the trend was not linear. These findings need to be validated further with other glucose control indicators (glycated hemoglobin), and may hint at a heterogenetic role of glucose level in FASP. Previous investigations primarily explored the association between LDL-C or T2DM with incident AS, while by delineating AS progression burden stratified by LDL-C joint T2DM among established AS, our study focused on this previously neglected population and found that there was an interplay between LDL-C and T2DM upon separate link with FASP [20, 39, 40]. This finding emphasizes the need for strict management of LDL-C serum levels and close monitoring of AS progression in T2DM-AS patients.

Our study attempts to partially fill a critical knowledge gap by understanding the relationship between LDL-C joint T2DM and FASP risk. Future large-sample epidemiological, multicenter, and clinical studies are required to validate the risk of FASP in T2DM patients with LDL-C. Whether aggressive LDL-C treatment could limit AS progression in T2DM patients merits further investigation.

### Strengths and Limitations

The major strengths of this investigation are its prospective cohort design and the enrollment of classical AS patients with comorbidities. The present study also has some limitations. First, the AS progression rate was predominantly evaluated using echocardiography ultrasound and did not apply major adverse cardiovascular events or surgical or transcatheter aortic valve replacement as outcomes. Second, our findings were observational, and the causal role of LDL-C combined with T2DM on FASP risk should be verified in further prospective intervention studies. Third, the echocardiographic examinations and the evaluation of AS status were performed by multiple physicians over the years, conceivably generating increased variability as we included patients already known for AS, and the lack of a core laboratory for echocardiograms limits the reproducibility of the study and may affect external validation. Finally, the sample size of our cohort may be seen as a limitation in demonstrating a potential association between LDL-C joint T2DM and FASP. Regardless, they compare well to the literature, and there was no trend suggesting that the absence of an impact of LDL-C joint with T2DM might be due to the small sample size or limited power.

## 5. Conclusions

Elevated LDL-C joint T2DM was associated with FASP. This investigation suggests that FASP may develop in T2DM patients with elevated LDL-C, highlighting the need for aggressive LDL-C management in these patient groups. Additional research is needed to validate this finding and explore the possible biological mechanism to improve the cardiometabolic management of T2DM and seek possible prevention for AS progression for this population.

## Data Availability

The datasets used and/or analyzed during the current study are available from the corresponding author on reasonable request.

## Abbreviations

T2DM, Type 2 diabetes mellitus; AS, aortic stenosis; CHD, coronary heart disease; LDL-C, low-density lipoprotein cholesterol; FASP, fast aortic stenosis progression; BMI, body mass index; TC, total cholesterol; UA, uric acid; AVA, aortic valve area; MPG, mean pressure gradients.

## Supplementary Material

Supplementary material associated with this article can be found, in the online version, at https://doi.org/10.31083/j.rcm2508276.
