# Supplementary material for: Low-Density Lipoprotein Cholesterol, Type 2 Diabetes and Progression of Aortic Stenosis: The RED-CARPET Heart Valve Subgroup Cohort Study
Source: Rev Cardiovasc Med. 2024 Aug 7;25(8):276. doi: 10.31083/j.rcm2508276 (PMC11366981; doi:10.31083/j.rcm2508276)
Supplement: Supplementary file 1 [file 2153-8174-25-8-276-s1.docx]

Supplementary table.1 Adjusted OR for fast progression rate of aortic stenosis

| Exposure | Unadjusted | | Partially adjusted model | | Fully adjusted model | |
| --- | --- | --- | --- | --- | --- | --- |
|  | OR (95%CI) | P value | OR (95%CI) | p value | OR (95%CI) | P value |
| **DM vs non-DM** | 2.33(1.06-5.13) | 0.035 | 2.31(1.02-5.22) | 0.045 | 2.42(1.00-5.584) | 0.050 |
| **LDL-C** |  |  |  |  |  |  |
| <2.15, mmol/L | Ref=1 |  | Ref=1 |  | Ref=1 |  |
| 2.15-3.14, mmol/L | 2.95(0.89-9.80) | 0.078 | 4.11(1.15-14.65) | 0.030 | 5.42(1.24-8.80) | 0.025 |
| ≥3.14, mmol/L | 3.31(1.04-10.56) | 0.043 | 5.84(1.64-20.87) | 0.007 | 11.90(1.55-15.65) | 0.017 |
|  |  |  |  |  |  |  |
| Partially adjusted model for age and gender; Fully adjusted model for age, gender, BMI, T2DM (excluded in T2DM comparison), TC, LDL-C (excluded in LDL-C comparison), Cr, HTN and CHD. OR, Odds ratio; CI, Confidence interval. Other abbreviation as in Table1. | | | | | | |
